# A rare case of isolated lung metastasis in the kidney

**DOI:** 10.1016/j.eucr.2021.101616

**Published:** 2021-02-26

**Authors:** B. Stoykov, P. Genov, I. Kirilov, K. Yanev, N. Kolev, V. Dunev

**Affiliations:** a University of Ruse “Angel Kanchev”, 8 “Studentska” str, 7000, Ruse, Bulgaria; b Medical University Pleven, “Georgi Kochev”8A str, 5800, Bulgaria; c Medical University Sofia, 1 “Georgi Sofiiski” str, 1431, Bulgaria

**Keywords:** Lung tumor metastasis, Kidney, Radical nephrectomy

## Abstract

Renal metastases are very rare condition in the clinical practice. The treatment is individualized and it depends on general status of the patient, involving of other organs and also the control of primary tumor. We are presenting a 64 years old woman, who had episodes of intermittent hematuria for two weeks. CT scan showed a large heterogeneous left kidney mass. The patient underwent open transabdominal radical left nephrectomy and the final diagnosis was isolated lung adenocarcinoma metastasis of the kidney.

## Introduction

Renal metastases are very rare condition in the clinical practice. Moreover, isolated metastases in the kidney are extremely rare. The primary localizations of the tumors that metastasize most common in the kidney are from the lung, breast, digestive tract, melanomas and lymphomas, but also some rare cases with other etiology have been reported. The treatment is individualized and it depends on general status of the patient, involving of other organs and also the control of primary tumor.

## Case presentation

We are presenting a 64 years old woman, who was admitted in our Urology Department with symptoms of recurrent left lumbar pain, irradiating towards inguinal area for one week. The patient also complains from nausea, without vomiting and weight loss. She had episodes of intermittent hematuria for two weeks. Upon physical examination no abdominal mass was palpated and the bowel sounds were diminished in all quadrants. All laboratory results were in normal ranges.

The patient has a history of right upper lobectomy for right lung adenocarcinoma (pT1aN0M0) before 1 year and the histological examination showed adenocarcinoma of the lung with no metastasis found in the dissected lymph nodes. The patient underwent chemotherapy and had 1 year of disease-free interval on postoperative follow-up examinations.

**Fig. 1:**
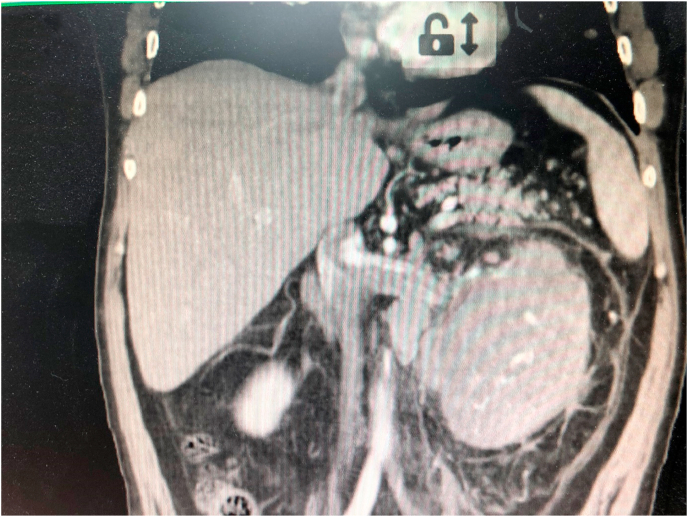
CT scan of left metastatic tumor of the kidney.

The contrast computed tomography (CT) scan of the chest and abdomen showed a large heterogeneous left kidney mass around 8 cm in diameter, engaging the upper pole and enhanced its density after the contrast no other pathological findings (Fig. 1). There were no other pathological findings on CT scan.

**Fig. 2:**
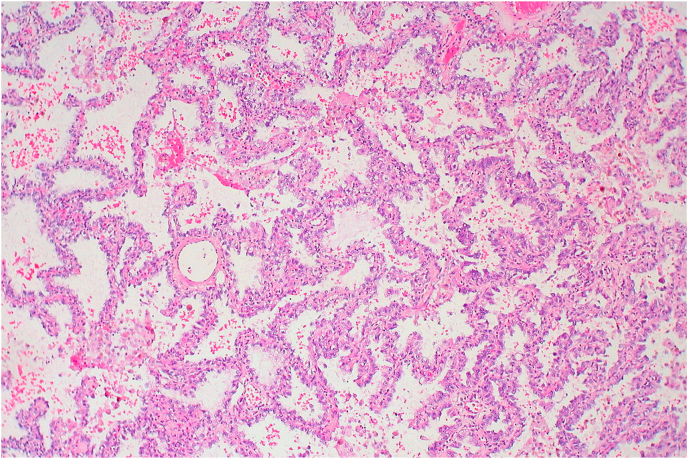
Histopathology image of lung tumor metastasis of the kidney.

The patient underwent open explorative transabdominal radical left nephrectomy with preliminary diagnosis of renal cell carcinoma. The whole tumor was removed using sharp and blunt dissection. Histopathology results showed a metastatic adenocarcinoma, which coincided with the histological findings of the previous surgery of the left lung (Fig. 2). The final diagnosis was isolated lung adenocarcinoma metastasis of the kidney.

## Discussion

Since the renal blood flow accounts for approximately 20% of cardiac output is assumed that the kidneys may be affected by hematogenous metastases.^1^ Cathy Zhou et al. retrospectively identified 151 patients diagnosed with a primary non-renal malignancy-renal metastasis Their results shows that the most common primary tumor sites were lung (43.7%), colorectal (10.6%), ENT (6%), breast (5.3%), soft tissue (5.3%), and thyroid (5.3%). Renal metastases were typically solitary (77.5%).^2^ In our case we have an isolated solitary metastasis from lung.

The most frequent symptoms of the metastatic tumors of the kidney are abdominal or flank pain, hematuria, weight loss, sweats and fever. Also in most of the patients with renal metastases there are no specific symptoms.^3^ Tomita M. et al. concludes that in their series of 64 cases of lung tumors with renal metastases, 50% have hematuria.^4^ In our case the patient has a typical symptoms of hematuria, weight loss and flank pain.

With regards to small cases reported in the literature, there is no clear guideline available for management of renal secondary tumors. Chemotherapy guided to the primary tumor can be useful, although results are usually poor. The role of solitary nephrectomy for metastasis in kidney has been unknown in detail because comparison of surgical versus nonsurgical treatment in the management of solitary metastatic disease in prospective randomized studies is almost impossible.^5^ However when the metastasis in the kidney is isolated and when we have a good control of primary tumor, the nephrectomy can give a long free survival interval of the disease, like in our case.

## Conclusions

Renal metastases are rarely seen in medical practice and that is way it is difficult to diagnose and choose the right treatment. Management of the disease is individualized and the nephrectomy can be a good option in selected cases.

## Declaration of competing interest

The authors declare that they have no competing interests.
